# Sleepiness is a signal to go to bed: data and model simulations

**DOI:** 10.1093/sleep/zsab123

**Published:** 2021-05-15

**Authors:** Tamar Shochat, Nayantara Santhi, Paula Herer, Derk-Jan Dijk, Anne C Skeldon

**Affiliations:** 1 Cheryl Spencer Department of Nursing, Faculty of Social Welfare and Health Sciences, University of Haifa, Haifa, Israel; 2 Surrey Sleep Research Centre, Faculty of Health and Medical Sciences, University of Surrey, Guildford, United Kingdom; 3 Department of Psychology, Faculty of Health and Life Sciences, Northumbria University, Newcastle Upon Tyne, United Kingdom; 4 UK Dementia Research Institute, Care Research & Technology Centre, at Imperial College London and the University of Surrey, Guildford, United Kingdom; 5 Department of Mathematics, Faculty of Engineering and Physical Sciences, University of Surrey, Guildford, United Kingdom

**Keywords:** sleepiness, sleep timing, sleep duration, sleep homeostasis, circadian regulation, mathematical model simulations

## Abstract

**Study Objectives:**

Assess the validity of a subjective measure of sleepiness as an indicator of sleep drive by quantifying associations between intraindividual variation in evening sleepiness and bedtime, sleep duration, and next morning and subsequent evening sleepiness, in young adults.

**Methods:**

Sleep timing and sleepiness were assessed in 19 students in late autumn and late spring on a total of 771 days. Karolinska Sleepiness Scales (KSS) were completed at half-hourly intervals at fixed clock times starting 4 h prior to participants’ habitual bedtime, and in the morning. Associations between sleepiness and sleep timing were evaluated by mixed model and nonparametric approaches and simulated with a mathematical model for the homeostatic and circadian regulation of sleepiness.

**Results:**

Intraindividual variation in evening sleepiness was very large, covering four or five points on the 9-point KSS scale, and was significantly associated with subsequent sleep timing. On average, a one point higher KSS value was followed by 20 min earlier bedtime, which led to 11 min longer sleep, which correlated with lower sleepiness next morning and the following evening. Associations between sleepiness and sleep timing were stronger in early compared to late sleepers. Model simulations indicated that the directions of associations between sleepiness and sleep timing are in accordance with their homeostatic and circadian regulation, even though much of the variance in evening sleepiness and details of its time course remain unexplained by the model.

**Conclusion:**

Subjective sleepiness is a valid indicator of the drive for sleep which, if acted upon, can reduce insufficient sleep.

**Statement of Significance**

Concerns about insufficient sleep are widespread and proposed remedies include flexible working hours and delayed work and school start times. An alternative remedy is to go to sleep earlier but our understanding of what determines the decision to go to sleep is limited. Here we show that higher levels of sleepiness in the evening associate with earlier bedtimes and longer sleep duration. To promote sufficient sleep and avoid delayed bedtimes, college students should appreciate sleepiness as a brain signal that it is time to go to sleep. We should all be encouraged to avoid conditions which hamper the expression of sleepiness in the evening.

## Introduction

According to recent accounts of William Dement’s magnificent contributions to sleep medicine and sleep research, he told Johnny Carson on The Tonight Show that “Sleepiness is a signal to go to bed.” https://med.stanford.edu/news/all-news/2020/06/william-dement-giant-in-field-of-sleep-medicine-dies-at-91.html

This was in the 1970s and since then sleepiness has often been studied as a consequence of insufficient sleep. Here, we investigate whether sleepiness is also a determinant of the time we go to bed.

Factors contributing to sleepiness have been investigated extensively. Sleep disordered breathing [1] sleep restriction and insufficient sleep [2] as well as circadian misalignment [3] all cause sleepiness.

Insufficient sleep and sleep timing variability contribute to sleepiness in a dose response manner. For example, in adolescents, daytime sleepiness increased following five nights of restriction to 5 h time in bed (TIB) [4], and following induced night-to-night variability in sleep duration [5]. Daytime sleepiness decreased linearly with increased TIB (7.0; 8.5; 10 h) [6]. In young adults, seven nights of restriction to 6 h TIB [7] and 14 nights of restriction to four and 6 h TIB [8] led to a proportionate increase in sleepiness. That sleepiness, measured subjectively or by polysomnography assessed sleep latency, is a sensitive measure of insufficient sleep, and homeostatic sleep pressure, is further supported by data showing that it was the outcome variable with the largest effect size in studies of SWS disruption [9] and sleep restriction [7, 10], and one of the earliest variables to respond [11] in experimental studies of sleep restriction. The relevance of self-reported sleepiness/alertness as a measure of brain state is further supported by the observation that, for a variety of tasks, it was a better predictor of performance than circadian melatonin phase, hours since awakening, or cumulative sleep loss [12].

Cumulative sleep loss in laboratory investigations is often quite severe, with reductions up to 4 h per night and it may therefore be unsurprising that sleep latency tests [6, 10] and self-reports [4, 5, 7, 8] show that participants experience increased sleepiness. Additional evidence for the sensitivity of subjective sleepiness may be derived from studies in the real world. In healthy individuals in their ecological setting, day-to-day variation in daytime sleepiness covaries with prior sleep duration, time of awakening and sleep quality. In one such study it was found that on average, for each hour of reduced sleep, sleepiness increased by 0.15 on the 9-point Karolinska Sleepiness scale (KSS) [13]. The range of variation in total sleep duration for 64% of this nonpathological adult sample was between 5:30 and 9:00 h, which equated to a modest yet highly significant change of 0.55 points in KSS. Although in this study earlier awakening and lower sleep quality were also independent and significant determinants of increased next-day sleepiness, their contribution was far weaker than the contribution of sleep duration.

Sleepiness is also under circadian control and this has been quantified in forced desynchrony [3, 14, 15], ultrashort [16] and 90-min [17, 18] sleep–wake cycle protocols. The circadian modulation of sleepiness is such that circadian sleepiness reaches its maximum late in the biological night, closely associated with the endogenous minimum of the core body temperature rhythm and declines during the biological day to reach a minimum shortly before habitual bedtime. This circadian phase has been referred to as the wake maintenance zone [19, 20], also known as the forbidden zone for sleep [16].

The combined effects of circadian phase and time awake on sleepiness have been documented in sleep deprivation studies in which both time awake and circadian phase were quantified. In these laboratory studies, sleepiness remained at rather stable levels throughout the waking day, until it increased just after the increase of nocturnal melatonin secretion [7, 14, 21]. In field studies the daily time course of sleepiness has been found to be u-shaped with moderate levels upon awakening, declining and reaching a minimum in the late afternoon and then increasing in the evening hours to levels that were higher than those that occurred upon awakening [2, 22]. Sleepiness is also affected by environmental and behavioral factors and individual characteristics such as caffeine intake [23], light exposure [24], workload and stress [25], as well as age [26] and chronotype [27]. These factors may act through their effects on both circadian and homeostatic processes or independently thereof.

The effects of circadian, homeostatic and other factors, have been captured in mathematical models most commonly by using derivatives of the two-process model of sleep regulation (e.g. [28, 29]) or the Phillips Robinson Model [30, 31].

In all of these approaches, sleepiness is viewed as an outcome measure. Here we explore to what extent sleepiness predicts the timing of sleep. According to this view, sleepiness is a brain signal coding for the drive for sleep behavior. If sleepiness is an indicator of the drive for sleep, which participants access and act on, then it should be a predictor of bedtime and evening-to-evening variation in sleepiness should predict night-to-night variation in sleep timing. An alternative possibility is that sleepiness is a signal which participants may choose to ignore or attempt to repress, by ingesting stimulants (e.g. caffeine) or engaging in stimulating activities (e.g. performing physical activity or watching a movie).The extent to which subjective sleepiness in the evening contributes to the decision to go to bed and associates with variation in sleep timing and subsequent sleep duration has not been studied longitudinally in an ecological environment, to our knowledge. Furthermore, most descriptions of the time course of sleepiness in the evening have had a rather limited temporal resolution [2] or were obtained in laboratory settings (e.g. [24]) which may have failed to detect variation in sleepiness on a short timescale or obtained a time course of sleepiness different from the time course in ecological settings.

From the point of view of the regulation of sleep by homeostatic and circadian factors [32, 33], as in the two-process model, we expect low levels of evening sleepiness not only to lead to later bedtimes, but also, because of the circadian regulation of sleep timing and duration [32, 33], we expect later bedtimes to be associated with shorter sleep durations. Later bedtimes should also lead to shorter sleep durations due to social constraints such as the need to get up for work or school [34]. Short sleep duration will lead to higher levels of sleepiness in the morning and continue to be elevated until the next evening.

Understanding this chain of events determined by homeostatic, circadian and environmental factors and disruption thereof may enable the design of interventions to prevent insufficient sleep and sleep irregularity.

The aim of the present study was to assess the extent to which this chain of events can be observed in sleepiness and sleep timing data collected in a naturalistic setting. In addition, we investigated the extent to which the day-to-day variation in sleepiness and sleep timing could be explained in a quantitative manner within the framework of existing theory by comparing our data to the predictions of a mathematical model for the circadian and homeostatic regulation of sleepiness and sleep timing and duration [35].

To address these aims requires that sleepiness is assessed at fixed clock times every evening and that sleepiness and sleep timing are recorded longitudinally. We chose to study this in young adults because this segment of the population has been reported to suffer extensively from insufficient sleep and associated consequences [36].

## Methods

### Ethics and participants

The research protocol received a favorable opinion from the University of Surrey Ethics Committee and was conducted in accordance with the principles of the Declaration of Helsinki. Written informed consent was obtained from all participants before starting any study related procedures. Twenty-three healthy young full-time university students, residing in university dormitories or off-campus housing, were recruited to the study. Exclusion criteria (all ascertained with validated health and sleep questionnaires) included working night shifts, having or being currently treated for a sleep disorder or for depression, having ophthalmologic or other neurological abnormalities, acute or chronic illness, taking medications on a chronic basis, particularly medications affecting the central nervous system, alcohol intake >14 units per week on average and consumption of more than four cups of caffeinated beverages (e.g. coffee, tea, cola) daily over the preceding one month. Of the 23 participants, two were removed due to incomplete diary measurements, one was removed due to nonreliable measurements, and one was removed due to incomplete actigraphy data. Of the remaining 19 participants (average age ± 1 *SD*: 18.9 ± 0.8 years, 12 females; Morningness-Eveningness Questionnaire score: 47.3 ± 6.3) who were entered into the analyses, one participant only completed self-report assessments in the late autumn.

### Study design and procedures

Detailed procedures are described in Shochat et al. [37]. Data were acquired during the late autumn of 2014 (November to December—starting at least 22 days after the change to standard time) and again in late spring (April–May 2015—starting at least 29 days after the change to daylight saving time) at the University of Surrey and its surroundings (Guildford, Surrey, 51.2362°N, 0.5704°W). Each segment included 3 weeks (during the semester) of daily monitoring of self-assessed evening and morning sleepiness levels, bed and wake times, and actigraphy-based sleep patterns using an Actiwatch-L. The Pittsburgh Sleep Quality Index (PSQI) was used to assess habitual sleep quality and timing [38].

**Figure 1.**
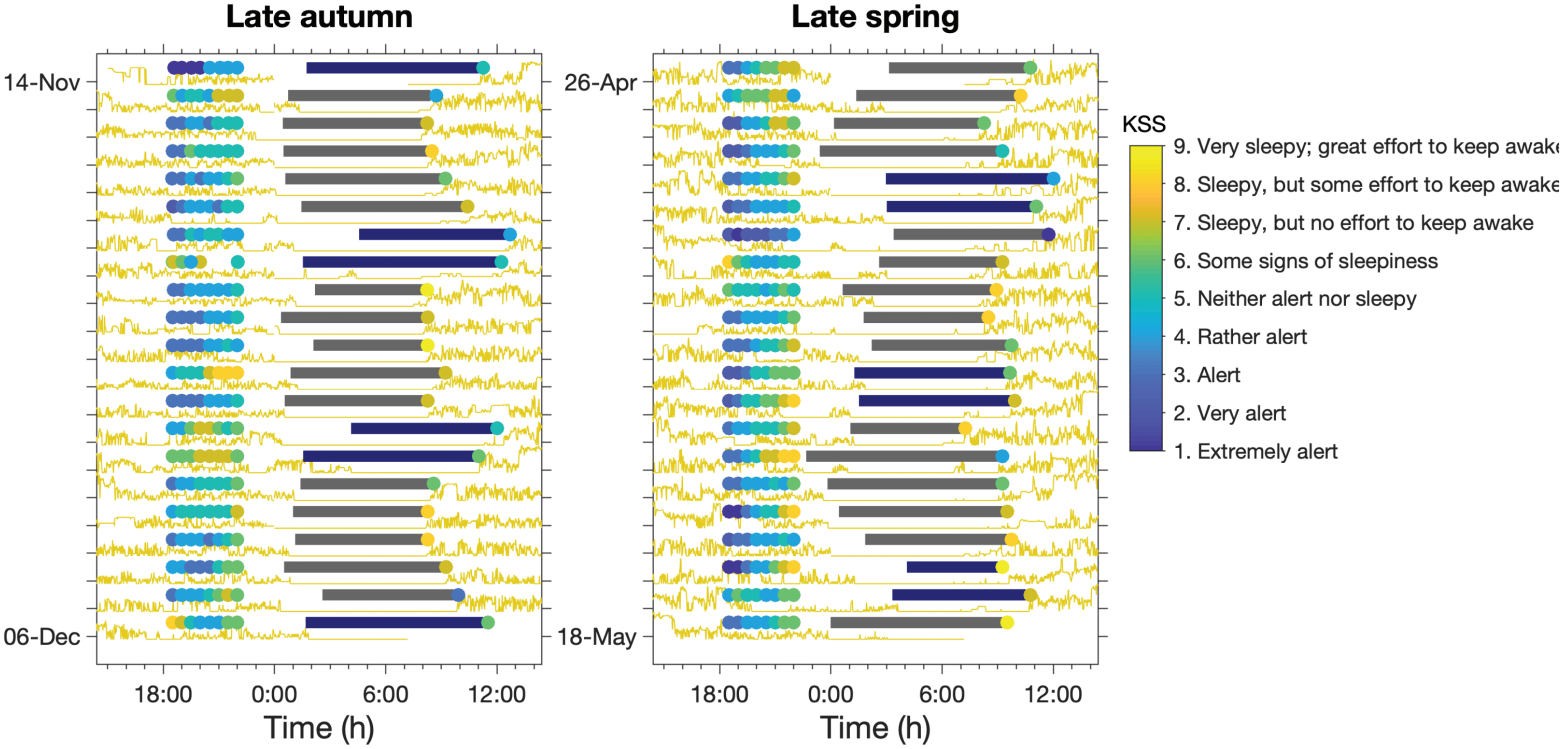
Evening and morning sleepiness, sleep timing and light exposure during late autumn and early spring in one participant. The horizontal bars indicate sleep timing. Each bar starts at sleep onset as defined by bedtime reported in the sleep diary and sleep latency as determined from actigrapy, and ends at wake time as reported in the sleep diary. Bars colored dark blue are weekend nights (Friday night to Saturday morning and Saturday night to Sunday morning). The circles before and after each “sleep period” are coloured according to the value on the Karolinska sleepiness scale (KSS). The yellow trace is light measured using an Actiwatch-L on a logarithmic scale.

For the data collection period, participants were requested to assess their present sleepiness level at half-hourly intervals for three and a half hours, starting 4 h prior to their individual habitual bedtime, leading to eight evening sleepiness assessments for each day. Participants were also asked about their reason to go to bed and whether or not they used an alarm clock. Each morning upon awakening, participants assessed their wake times, their level of morning sleepiness as well as their previous night’s bedtime. For evening and morning sleepiness assessments, participants recorded the exact time of each assessment. The protocol is illustrated in Figure 1, where the results for a single participant are shown.

### Assessment tools

#### Sleepiness levels

Sleepiness was monitored with the Karolinska Sleepiness Scale (KSS), a 9-point measure of subjective sleepiness. Participants were requested to rank their level of sleepiness in the immediately preceding period, from 1: “extremely alert,” to 9: “very sleepy, great effort to keep awake.” The KSS is widely used and has been validated with respect to objective measures of sleepiness [2, 39], such as slow rolling eye movements and alpha-theta power density during waking [39], and found to compare well with the multiple sleep latency test (MSLT) [26]. Here we used the first reported evening KSS (evKSS1), the eighth reported evening KSS (evKSS8), the average over all evening assessments (evKSSav), and the morning KSS (moKSS). We elected to use the KSS rather than objective measures of sleepiness such as the MSLT since KSS is a measure that is readily available to people in their normal daily lives.

#### Nightly sleep timing and duration

The Karolinska Sleep Diary (KSD) was used for assessing daily sleep patterns [40]. This self-report sleep diary provides information on self-selected daily bed- and wake times. Sleep duration was computed as the number of minutes between self-reported bed and wake time, minus the number of minutes of sleep latency as recorded by actigraphy (Actiwatch-L, CamNtech Ltd).

#### Use of an alarm clock

The KSD includes an item on the use of an alarm clock: “*Did your alarm clock wake you up?”* which allowed for a narrative answer. Since the narrative answers from the late autumn indicated that students sometimes set an alarm but woke before it, for the spring assessments the single question was replaced by two yes/no questions, “*Did your alarm clock wake you up?”* “*Did you set an alarm clock?.”*

#### Reason for bedtime

During the late spring assessments only, participants were asked about the reason for going to bed: *“Why did you go to sleep at the time that you did?.”* Responses included *“sleepy,” “tired,” “needed to wake early,” “usual bedtime”* and *“other.”* Participants could select more than one category.

#### Bedtime category

Participants were divided into early, intermediate and late bedtimes based on their weekday bedtimes during the autumn and using the following cutoffs: early (E): ≤23:30 (*n* = 8); intermediate (I): between 23:30 and 01:00 (*n* = 4), and late: (L): ≥01:00 (*n* = 7), see Shochat et al. [37].

### Statistical analyses

Analyses were performed with SAS version 9.4, with significance set at 0.05 and with MATLAB R2019a [41]. The R package was used to compute repeated measures correlations (RMcorr) [42].

Participants differed in absolute measures of sleepiness and sleep timing: for averages and standard deviations of the average and median values of sleep timing and sleepiness variables per participant, over all assessments and by season, see Table 1. We used two approaches to account for “subject effects.” In the first approach, we ran mixed models to assess the association between evening KSS (evKSS1, evKSS8, evKSSav as independent variables – IVs) and bedtime (as a dependent variable – DV), bedtime (IV) and sleep duration (DV), sleep duration (IV) and next morning sleepiness (moKSS, DV), and morning sleepiness (IV) and next evening sleepiness (evKSS8, DV) with participant as a random effect. Next, we ran the models and assessed interactions with season and bedtime category as fixed effects. For all mixed models, compound symmetry was used as the covariance structure, and the Kenward–Roger method was used to estimate the true degrees of freedom for the tests of significance [43]. Please note that since “participant” was included as a random effect, i.e. the intercept of the regression of dependent and independent variables varies between participants, effects of independent variables on dependent variables indicate that intraindividual variations in these variables are associated.

**Table 1.** Sleep timing, sleep duration and sleepiness

		Average ± SD of average / participant (*n*)	Average ± SD of median / participant (*n*)	Range (average subject)
Bedtime	Total	24:45 ± 1:17 (19)	24:35 ± 1:22 (19)	23:00–3:16
	Late Autumn	24:47 ± 1:10 (19)	24:31 ± 1:17 (19)	22:51–3:44
	Late Spring	24:47 ± 1:31 (18)	24:36 ± 1:35 (18)	22:04–3:01
Wake time	Total	9:08 ± 0:49 (19)	8:58 ± 0:50 (19)	7:42–10:44
	Late Autumn	9:08 ± 0:44 (19)	8:51 ± 0:45 (19)	8:07–10:41
	Late Spring	9:10 ± 1:07 (18)	9:06 ± 1:00 (18)	7:17–11:04
Sleep duration	Total	7:49 ± 0:56 (19)	7:47 ± 0:58 (19)	5:15–9:19
	Late Autumn	7:49 ± 1:07 (19)	7:42 ± 1:11 (19)	3:50–9:14
	Late Spring	7:47 ± 0:54 (18)	7:46 ± 0:59 (18)	5:57–9:06
evKSS 1	Total	3.98 ± 1.09 (19)	3.89 ± 1.29 (19)	2.45–6.15
	Late Autumn	4.01 ± 1.19 (19)	3.74 ± 1.24 (19)	2.38–6.57
	Late Spring	3.89 ± 1.07 (18)	3.86 ± 1.23 (18)	2.19–5.70
evKSS8	Total	6.78 ± 0.81 (19)	6.97 ± 0.89 (19)	5.56–8.09
	Late Autumn	6.87 ± 0.98 (19)	7.08 ± 1.14 (19)	5.57–8.33
	Late Spring	6.61 ± 0.66 (18)	6.63 ± 0.90 (18)	5.52–7.80
evKSSav	Total	5.30 ± 0.84 (19)	5.34 ± 0.88 (19)	4.12–6.90
	Late Autumn	5.29 ± 0.86 (19)	5.26 ± 1.05 (19)	4.07–6.96
	Late Spring	5.23 ± 0.74 (18)	5.19 ± 0.75 (18)	4.10–6.89
moKSS	Total	5.64 ± 1.37 (19)	5.50 ± 1.55 (19)	3.83–8.49
	Late Autumn	5.68 ± 1.34 (19)	5.68 ± 1.52 (19)	3.81–8.45
	Late Spring	5.50 ± 1.44 (18)	5.44 ± 1.62 (18)	3.67–8.52

Averages and medians were calculated for each participant and then averaged across participants over all study days (Total) and by season (Late Autumn, Late Spring). evKSS1: first evening KSS, evKSS8: eighth evening KSS, evKSSav: mean evening KSS, moKSS: morning KSS.

To test the robustness of the results of the mixed model, which assumes that variables are normally distributed we also use a nonparametric approach. In this approach “participant” effects were controlled for by expressing observations as deviations from participants’ median scores. Specifically, for each of the following: evKSS1, evKSS8, evKSSav, moKSS, bedtime, and sleep duration, and per participant, we computed the participants’ median score per season, and then computed their daily deviations from that median score. Thus, positive deviations (higher scores than the median) indicated higher sleepiness, later bedtimes, and longer sleep duration than usual, and negative deviations (lower than the median) indicated lower sleepiness, earlier bedtimes, and shorter sleep duration than usual. To assess the robustness of the effects, we used nonparametric Spearman rho correlation coefficients and tested associations between deviations from median evening sleepiness and bedtime, bedtime and sleep duration, sleep duration and morning sleepiness, and morning sleepiness and subsequent evening sleepiness. These associations were tested over all observations, as well as by season (late autumn, late spring), by bedtime category (early, intermediate, late), and by day-of-the-week (Sunday-Saturday). A False Discovery Rate (FDR) adjustment was applied to control for multiple correlations. We also ran repeated measures correlations [42], which adjust for nonindependence due to repeated measurements. To test whether two nonparametric correlations were significantly different, we performed a Fisher r to z transformation as implemented in https://www.psychometrica.de/correlation.html. We compared differences in correlations between late autumn and late spring, between early, intermediate and late bedtime categories, between days of the week, and between evKSS1 and evKSS8. Finally, we ran linear regressions on the values expressed as deviations to estimate the slope. We used the slopes of the linear regressions for comparison with the mixed model and mathematical simulation approaches.

### Model simulations

**Figure 2.**
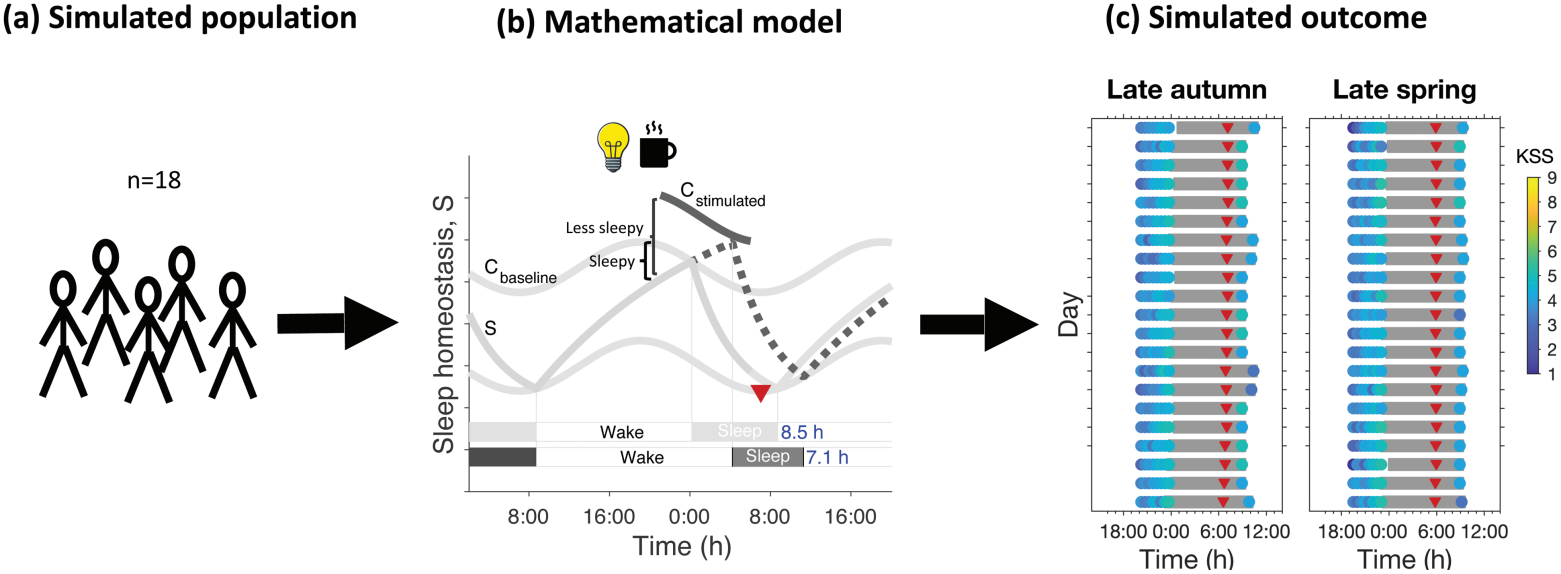
Schematic representation of the mathematical modeling of the experiment. (a) A population consisting of 18 model participants was constructed in line with the number of participants for the field protocol. Each participant was assigned a different intrinsic circadian period and light exposure pattern. (b) A two-process-like mathematical model that included homeostatic sleep pressure (S) and circadian rhythmicity (C) along with light and social constraints was then used to simulate each participant. Sleepiness was modeled as proportional to the distance between S and the upper threshold of C, such that sleepiness increases when the distance between the upper threshold and S becomes smaller. Day-to-day variation in sleep timing and sleepiness was modeled by varying the position of the upper threshold. Thus a higher upper threshold (dark gray line), as for example induced by direct effects of light or caffeine, led to lower sleepiness. (c) Model outputs included sleep timing (gray bars), measures of evening and morning sleepiness (color coded circles) and circadian phase (red triangles).

To investigate whether the associations observed in the data were consistent with current mathematical models for the homeostatic and circadian regulation of sleep we simulated the protocol, as summarized in Figure 2, using a quantitative mathematical model that includes sleep homeostasis, circadian rhythmicity, the effect of light on circadian phase and social constraints [35]. Our aim was to see if by making reasonable assumptions on light, intrinsic circadian period and “noise” we could capture the observed distributions and day-to-day variation in sleep timing and sleepiness. This is distinct from previous approaches in which fitting to individual participant sleep schedules has been carried out with, for example, phase optimized to best fit KSS data [29].

The mathematical model used was an adapted version [35] of a neuronal model [44] which describes sleep regulation as a flip-flop switch between the firing of wake-promoting and sleep-promoting neurons [45] combined with a Kronauer-style model [46] that models how light input via the eye determines circadian phase. Model inputs were light exposure and intrinsic circadian period. Model outputs included, circadian phase, sleep timing and “sleep drive.” Simulations were carried out using MATLAB R2019a [41].

#### Modeling sleepiness using data from a laboratory protocol

Since KSS is not a direct output of the neuronal model, we first developed a model that related sleep drive to KSS using laboratory data from a sleep restriction/sleep extension with subsequent sleep deprivation protocol [7, 47]. In the neuronal model sleep drive is the two-process model equivalent of taking the difference between the upper threshold—which models the circadian alerting effect—and process S which models the sleep homeostat [48].

The laboratory protocol included repeated KSS measurements across seven days of sleep restriction followed by 40 h of sleep deprivation. In this protocol individual differences in circadian phase were minimized by requiring the participants to maintain a regular sleep schedule prior to the laboratory segment. During the laboratory segment sleep was scheduled in accordance with the participants’ habitual schedule. Full details of the protocol are given in [7]. We simulated the full 12-day laboratory protocol, and the prelaboratory period for both sleep extension and sleep restriction conditions for an average participant. Since our model predicts spontaneous sleep timing, as in the laboratory study we aligned the simulated protocol to habitual sleep time. The sleep drive was evaluated at the appropriate average recorded times for each KSS measurement. Linear regression was used to relate sleep drive and the measured KSS values.

Previous authors have used a similar neuronal model and modeled sleepiness as a linear combination of homeostatic sleep pressure and circadian wake propensity [49]. Our motivation for using the particular neuronal model discussed here and therefore creating a new model for sleepiness is that, unlike other versions, it has been adapted to fit population averages for sleep duration and timing [35].

#### Modeling light input

Within the model, light exposure, sleep timing and intrinsic circadian period determine circadian phase. For simulating the field protocol we constructed a light profile that varied in maximum daytime intensity from day-to-day. The maximum daytime intensity was drawn from a log normal distribution and average values for light exposure patterns were motivated by average observed values [37]. Seasonal differences were modeled by selecting the average for the log normal distribution to be greater and the “natural” photoperiod as longer in the late spring than in the late autumn.

#### Modeling intraindividual variation in sleep timing and circadian phase

We have previously shown that neuronal models can quantitatively replicate average sleep timing and duration across the lifespan [50]. In the data collected from the student population, sleep duration sometimes varied by more than 5 h from one day to the next. To capture such large variations, we included a short-term random process that results in changes in sleep drive on the timescale of a few minutes. The timescale of a few minutes was slow enough that the model may still be thought of as a two-process-like model [31]. The short-term random process was then effectively equivalent to adding noise to the circadian thresholds of the two-process model, without changing their phase [51]. In the context of the two-process model, this approach has previously been used to model sleep duration in depression [52]. Taking the view that the “noise” is primarily a result of reactions to events that happen during wake e.g. boredom, excitement, light, etc., we kept the thresholds at their baseline levels during sleep.

#### Modeling social constraints

Social constraints were modeled by allowing “participants” to exert control over the times they were awake [35, 51], which at some times of day required “effort” [30]. Responses to the questions on setting and waking with an alarm indicated that on Monday to Friday students set an alarm 88% of the time. At the weekend, consistent with fewer educational commitments, they set an alarm 48% of the time. For the purposes of modeling, we made the assumption that an alarm was set only on weekdays, that everyone set the same alarm time and that students had to be awake from the time the alarm was set until 19:00 h in the evening.

Model outputs include sleep onset and offset times but not bedtime. For comparison with the field data, we have assumed that deviations in model sleep onset times are comparable with deviations in bedtime.

#### Modeling between-participant differences

In line with the number of students that took part in the actual study, we constructed a population of 18 “students.” We set parameters in the model so that each “student” had the same baseline sleep duration but had different intrinsic circadian periods drawn from a normal distribution with average 24.15 h ± 0.20 (*SD*) h in accordance with the observed distribution [53].

Full details of the equations, modeling assumptions and parameters are given in the Supplementary Material.

## Results

### Data

Participants completed a high proportion of the scheduled KSS assessments and sleep diary entries (98% of KSS assessments, 98% of bedtimes, 99% of wake times).

Participants were instructed to make their first assessment 4 h before their habitual bedtime, as determined from the PSQI which they completed at the start of the study in the late autumn. Using “habitual bedtime” derived from the diary bedtime data, showed that participants completed the evKSS1 measurement 4.22 ± 1.20 h before their median bedtime in the late autumn and 4.18 ± 1.75 h before their median bedtime in the late spring, see Supplementary Figure S1, a and c.

From day-to-day, participants were highly regular in the timing of their evening KSS assessment, as illustrated for one participant in Figure 1. Across all participants, the timing of the first evening KSS, evKSS1, deviated from the participant average by less than 7.5 min for 94% of all assessments in the late autumn and 96% in the late spring, see Supplementary Figure S1, b and d. Thus, the PSQI derived habitual bedtime was close to the subsequently observed bedtimes and KSS assessments were completed in good compliance with the instructions.

**Figure 3.**
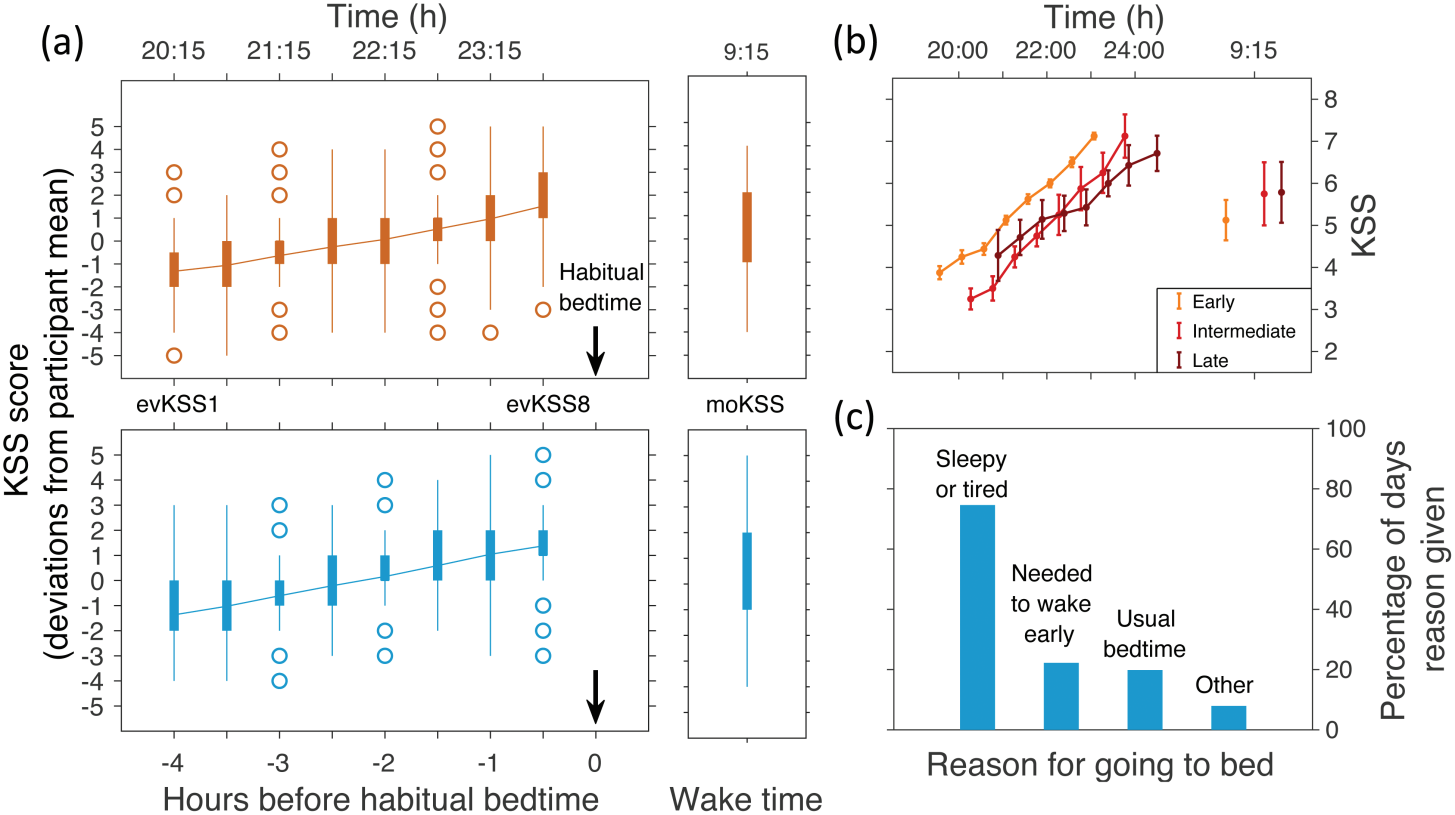
Time course of sleepiness during the evening and reported reasons for going to bed. Panel (a) Sleepiness across eight Karolinska sleepiness scale (KSS) assessments in the evening (evKSS1-8) and in the morning (moKSS) plotted at the average time of the assessment in local clock time and hours before habitual bedtime for the late autumn (upper panel) and the late spring (lower panel) respectively. To illustrate both the time course of sleepiness and the within participant variation, evKSS1-8 and moKSS were expressed as deviations from the overall KSS median score for each individual participant separately for each season. Panel (b) Time course of average KSS scores for evKSS1-8 and moKSS, for participants in early, intermediate and late bedtime categories and plotted at the average local clock times across observations. Vertical bars reflect the between participant standard error. Panel (c) Reported reasons for going to bed. Participants could select more than one option, so the bars sum to more than 100%. The reason for going to bed was only asked in the late spring.

As suggested by the single participant shown in Figure 1, sleepiness increased across the evening. This is further shown in Table 1 and in Figure 3. In Figure 3a boxplots for the deviation from the participant median KSS score of evKSS1 through to evKSS8 and the subsequent morning KSS, moKSS, are shown. Across the eight evening assessments, sleepiness increased by three points in both late autumn and late spring and dropped by one point overnight. This corresponded to a median increase across the evening from four to seven points on the KSS scale, with significant differences between the three bedtime categories (*F*(2, 5912) = 128.65, *p* < 0.001). Late sleepers had a less steep gradient than early and intermediate sleepers: per hour, KSS increased by 0.43 points in early, 0.51 points in intermediate, and 0.30 points in late sleepers (paired comparisons: early vs. intermediate *t* = 3.24, *p* < 0.0012; early vs. late: *t* = 11.13, *p* < 0.001; intermediate vs. late *t* = 13.65, *p* < 0.003, Figure 3b). Those in the early bedtime category were 0.36 KSS points less sleepy in the morning compared to the intermediate and late groups, however, group differences were nonsignificant (*F*(2,15.7) = 0.14, *p* > 0.87).

**Figure 4.**
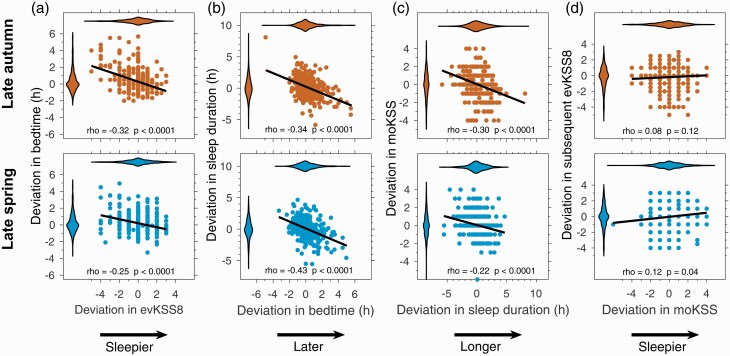
Associations between intraindividual variation in evening sleepiness and subsequent bedtime, bedtime and subsequent sleep duration, sleep duration and the following morning KSS, morning KSS and the following evening KSS. For each panel the distributions of the variables are shown using violin plots, where the violin plot for the variable plotted on the horizontal axis is at the top of the panel and the violin plot for the variable plotted on the vertical axis is to the left of the panel. The violin plots show that most of the distributions have long tails. All variables are expressed as deviations from the participant median value. The Spearman rho correlation coefficients and associated adjusted p values are shown in each panel.

Intraindividual day-to-day deviations in evening sleepiness (evKSS1, evKSS8, evKSSav) and bedtimes covered a large range. Specifically, deviations in evKSS8 from participant median value were from –5 to +3 points, covering almost the entire range of the 9-point KSS scale (violin plot, Figure 4a). Deviations in bedtimes ranged from nearly 5 h before to nearly 6 h after median bedtime (violin plot, Figure 4b).

Sleepiness was the leading reason that participants gave for going to bed. On 75% of the study days, participants selected that they were either sleepy (44%), tired (58%) or both sleepy and tired (27%), see Figure 3c. For 13 of the 18 participants (72%), feeling sleepy or tired was the main reason given for going to bed.

In order to assess the degree to which the day-to-day variation in evening sleepiness was correlated with subsequent bedtime and the “homeostatic regulatory chain” of reduced sleepiness leading to later bedtime, shorter sleep duration, higher subsequent morning KSS and higher subsequent evening sleepiness, we then considered successive associations for each stage in the above-mentioned chain of events. The results are summarized in Tables 2–4 and Figure 4.

**Table 2.** Mixed model associations between evening sleepiness and subsequent bedtime, bedtime and subsequent sleep duration, sleep duration and subsequent morning sleepiness, and morning sleepiness and subsequent evening sleepiness

	*F* (df)	*p*	*b*	95% CI
evKSS1 –Bedtime *	7.99 (1,753)	0.005	−6.82 (min/KSS point)	−11.56, −2.09
evKSS8 –Bedtime*	84.79 (1,686)	<0.0001	−18.43 (min/KSS point)	−22.35, −14.50
evKSSav –Bedtime*	52.06 (1,750)	<0.0001	−19.56 (min/KSS point)	−24.86, −14.23
Bedtime –Sleep duration	177.63 (1, 709)	<0.0001	−32.71 (min/h)	−37.34, −27.62
Sleep duration –moKSS	42.04 (1, 696)	<0.0001	−0.20 (KSS point/h)	−0.26, −0.13
moKSS -evKSS8	7.27 (1, 571)	0.007	0.11 (KSS point/KSS point)	0.03, 0.18

*Differences by bedtime category (see text and Table 4). evKSS1: first evening KSS; evKSS8: last evening KSS; evKSSav: average evening KSS, BT: bedtime (h), SD: sleep duration (min), moKSS: morning KSS.

### Evening sleepiness correlated with bedtime

According to both the mixed model parametric analysis and the nonparametric and repeated measures correlation analyses, evening sleepiness was significantly associated with subsequent bedtime (Tables 2 and 3, Figure 4), so that higher than usual evening sleepiness was associated with going to bed earlier than usual. For every one point increase in evKSS1, bedtime advanced by 7 min; for every one point increase in evKSS8 bedtime advanced by 18 min; and for every one point increase in evKSSav, bedtime advanced by 20 min. There was a significant interaction between evKSS8 and season on bedtime, with a stronger association between evKSS8 and bedtime in late autumn than in late spring (*F*(1,672) = 4.75, *p* = 0.03). Each one point increase in evKSS8 was associated with a 24 min advance in bedtime in late autumn and a 15 min advance in bedtime in late spring (a difference of 9 min). There was a significant interaction between evKSS8 and bedtime category, with a stronger association between evKSS8 and bedtime in early sleepers compared to late sleepers (*F*(2,683) = 2.91, *p* = 0.055; near significance). Each one point increase of evKSS8 was associated with a 23 min advance in bedtime for early sleepers, and a 12 min advance in bedtime for late sleepers (a difference of 11 min). Similarly, the association between evKSSav and bedtime was also stronger in early sleepers compared to late sleepers (*F*(2,734) = 3.80, *p* = 0.023); so that each one point increase in evKSSav was associated with a 27 min advance in bedtime for early sleepers, and a 10 min advance in bedtime for late sleepers (a difference of 17 min). A significantly stronger associations between evKSS8 and bedtime for early than late bedtime category was also found in the nonparametric correlations (Table 4).

**Table 3.** Correlations (Spearman rho and repeated measures) between intra-individual variation in evening sleepiness and subsequent bedtime, bedtime and subsequent sleep duration, sleep duration and subsequent morning sleepiness, and morning sleepiness and subsequent evening sleepiness, over all nights

	Spearman rho			Repeated Measures Correlations			
	*n*	rho	p*	df	r_rm_	*p*	95%CI
evKSS1 –Bedtime	768	−0.12	0.0014	736	−0.11	0.0026	−0.18, −0.04
evKSS8 –Bedtime	707	−0.28	<0.0001	675	−0.33	<0.0001	−0.40, −0.26
evKSSav–Bedtime	768	−0.22	<0.0001	736	−0.26	<0.0001	−0.33, −0.19
Bedtime –Sleep duration	729	−0.38	<0.0001	709	−0.43	<0.0001	−0.49, −0.37
Sl eep duration – moKSS	716	−0.26	<0.0001	696	−0.23	<0.0001	−0.30, −0.16
moKSS - evKSS8	664	0.10	0.008	632	0.09	0.03	0.01, 0.17

All variables were expressed as deviations from the median per participant per season and then entered into the correlation analysis.

*p values are corrected according to the False Discovery Rate (FDR) adjustment. evKSS1: first evening KSS, evKSS8: eighth evening KSS, evKSSav: mean evening KSS, moKSS: morning KSS; rrm: repeated measures correlation.

**Table 4.** Mixed models and correlations (Spearman rho and repeated measures) between intra-individual variation in evening sleepiness and subsequent bedtime, bedtime and subsequent sleep duration, sleep duration and subsequent morning sleepiness, and morning sleepiness and subsequent evening sleepiness, by bedtimecategory (early, intermediate, late)

		Mixed models		Spearman rho			Repeated Measures Correlations			
		*b* ^#^	95%CI	*n*	rho	p*	df	*r*_rm_	*p*	95%CI
evKSS8 - Bedtime	Early	−22.39	−28.00, −16.79	276	−0.37	<0.0001	255	−0.43	<0.0001	−0.53, −0.33
	Intermediate	−17.19	−25.96, −8.42	163	−0.28	0.0008	158	−0.33	<0.0001	−0.43, −0.18
	Late	−11.59	−18.41, −4.77	268	−0.21**	0.0006	260	−0.20	0.001	−0.32, −0.08
Bedtime- Sleep duration	Early	−32.63	−40.43, −24.84	284	−0.48	<0.0001	275	−0.50	<0.0001	−0.58, −0.40
	Intermediate	−33.19	−44.56, −28.81	162	−0.36	<0.0001	157	−0.41	<0.0001	−0.53, −0.27
	Late	−33.52	−41.75, −25.28	283	−0.34**	<0.0001	275	−0.38	<0.0001	−0.48, −0.28
Sleep duration – moKSS	Early	−0.21	−0.31, −0.10	278	−0.25	<0.0001	269	−0.21	0.0004	−0.32, −0.10
	Intermediate	−0.12	−0.25, 0.01	161	−0.15	0.08	156	−0.13	0.12	−0.28, 0.03
	Late	−0.23	−0.32, 0.14	277	−0.35***	<.0001	269	−0.33	<0.0001	−0.43, −0.22
moKSS- evKSS8	Early	0.07	−0.06, 0.20	260	0.09	0.149	239	0.05	0.42	−0.08, 0.18
	Intermediate	0.21	0.06, 0.35	155	0.17	0.046	150	0.25	0.002	0.09, 0.40
	Late	0.06	−0.08, 0.20	249	0.10	0.129	269	0.01	0.83	−0.11, 0.14

Spearman rho was expressed as deviations from the median per participant per season. Mixed models and repeated measures correlations based on the raw data.

#Slopes (beta values) represent min/KSS point (evKSS8-bedtime), min/h (Bedtime -Sleep duration), KSS/h (Sleep duration-moKSS) and KSS/KSS (moKSS-evKSS8).

*p values are corrected according to the False Discovery Rate (FDR) adjustment.

**Based on Fisher z, correlation for the late bedtime category is significantly weaker than the early bedtime category (*z* = 1.9, *p* = 0.03).

***Based on Fisher z, correlation for the late bedtime category is significantly stronger than the intermediate bedtime category (*z* = 2.1, *p* = 0.02). evKSS8: eighth evening KSS; moKSS: morning KSS; **r**_**rm**_:repeated measures correlation.

By day-of-the-week, correlations between last evening sleepiness (eKSS8) and bedtime were significant on all nights except Thursday (Supplementary Table S1).

### Bedtime correlated with subsequent sleep duration

According to the mixed model approach, bedtime was associated with subsequent sleep duration, so that for every hour earlier bedtime, sleep duration increased by 33 min. These associations did not differ by season or by bedtime category. Deviations in sleep duration from participant’s median ranged from –5.9 to +8.1 h (Figure 4b). The nonparametric correlations confirmed that deviations in sleep duration were negatively correlated with deviations in bedtime (Table 3); and these associations were further confirmed using the repeated measures correlations approach and the raw data. These correlations were significant over all observations, and both in late autumn and in late spring, with no seasonal differences in the strength of the associations (not shown). Correlations were significant for early, intermediate and late bedtime categories, but stronger in early than late bedtime categories (Table 4). Correlations were significant on all nights of the week (Supplementary Table S1).

### Sleep duration correlated with subsequent morning sleepiness

Based on the mixed model approach, sleep duration was associated with moKSS, so that for every hour increase in sleep duration, moKSS decreased by 0.20 points. These associations did not differ by season or by bedtime category. The nonparametric and repeated measures correlation analyses also showed that deviation in sleep duration was negatively correlated with deviation in morning sleepiness (moKSS). These correlations were found over all observations (Table 3), in late autumn and in late spring, with no seasonal differences in the strength of the associations (not shown). Correlations and violin plots are shown in Figure 4c and illustrate the wide range of sleepiness values in the morning (deviation from participant median, –6 to +4 KSS points).

The non-parametric correlations were significant in early and late sleepers but not in the intermediate sleepers, with a stronger association in late than early sleepers (Table 4). By day, these correlations ranged between *r*_s_ = –0.18 (Thursday) to *r*_s_ = 0.35 (Saturday) and did not differ in strength between consecutive days of the week (Supplementary Table S1).

### Morning sleepiness correlated with subsequent evening sleepiness

Based on the mixed model approach, moKSS was associated with subsequent evKSS8; so that for every one point increase in moKSS, evKSS8 increased by 0.11 points. These associations did not differ by season or by bedtime category. The nonparametric and repeated measures correlation approaches also indicated that deviation in morning sleepiness positively correlated with the deviation in subsequent evening sleepiness. The correlation between moKSS and evKSS8 was significant in late spring but not in late autumn, but the strengths of the correlations in late spring and in late autumn were not significantly different (Figure 4d). The correlation between moKSS and next evening evKSS8 was significant only for the intermediate bedtime category, but the strengths of the correlations did not differ between early, intermediate and late sleepers (Table 4). By day of the week, these associations did not reach significance (Supplementary Table S1).

### Scaling model output to KSS by simulating a laboratory study of the effects of sleep restriction and total sleep deprivation

**Figure 5.**
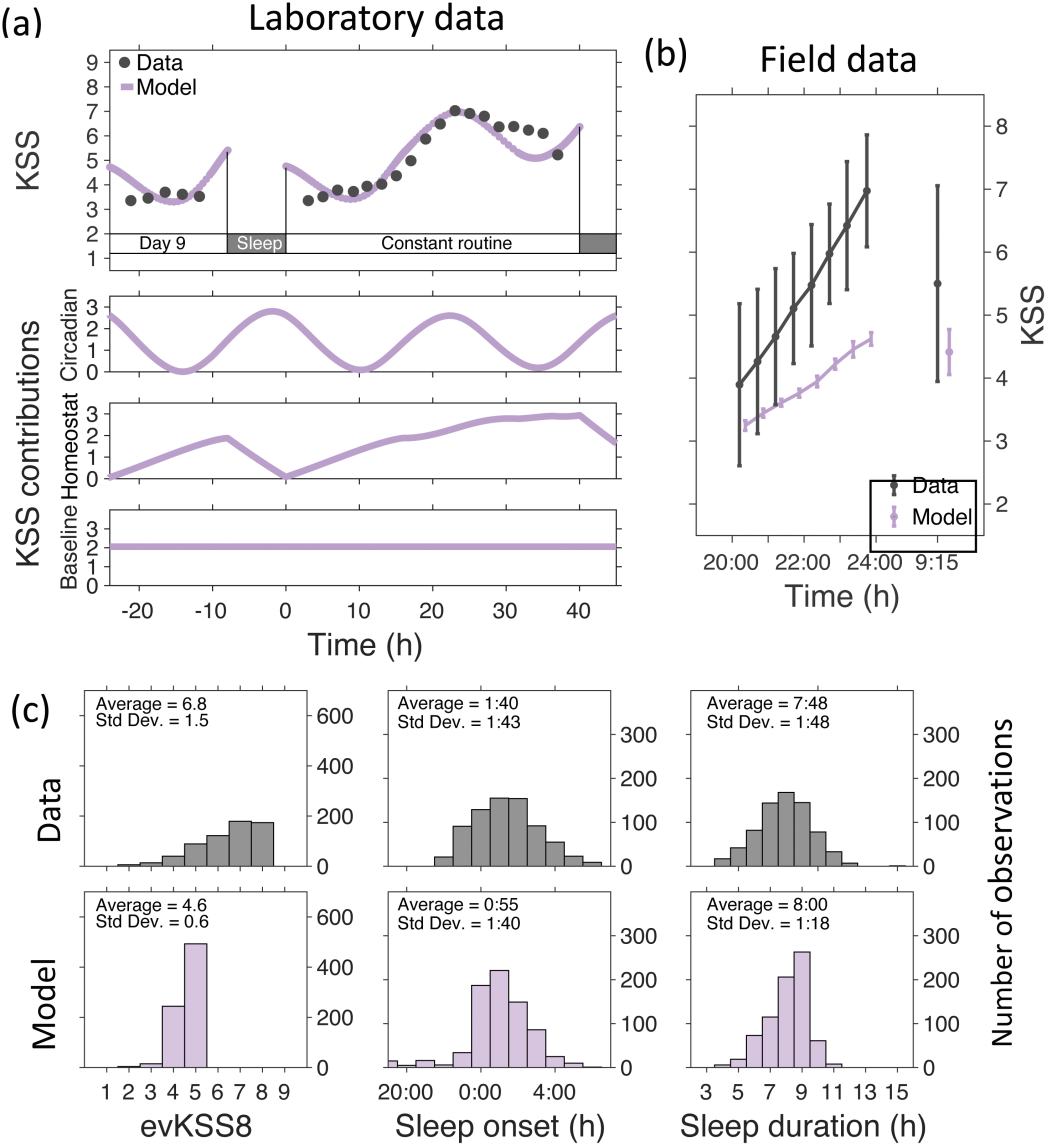
Model fitting to laboratory data and comparison between simulations and data for KSS, bedtime and sleep duration. Panel (a) Fitted KSS time course using the mathematical model and data from a laboratory sleep extension / sleep restriction followed by 40 h of sleep deprivation study [7]. Only days 9–11 of the 12-day protocol are shown. The modeled KSS time course consisted of circadian, homeostatic and baseline contributions which are also shown. Panel (b) Time course of average KSS scores for evKSS1-8 and moKSS, for participants in the field study and for the simuated protocol. Data points are plotted at the average local clock times across observations with the exception of the simulated moKSS which has been displaced horizontally for clarity. Vertical bars reflect the between participant standard deviation. Panel (c) Histograms of the observed and simulated eighth evening KSS (evKSS8) assessment, sleep onset and sleep duration. Data for all participants and all days are included. The labels for all bins are centered. Differences between observed and simulated KSS are in part a consequence of known differences between field and laboratory measurements [13].

Sleep drive (*D*_v_) from the mathematical model was highly correlated with the KSS data in the sleep extension / sleep restriction with subsequent total sleep deprivation protocol (Supplementary Figure S5b, rho = 0.73, *p* < 0.0001). Linear regression between KSS and sleep drive *D*_v_ resulted in the relationship


$$\text{KSS}=0.79D_{v}+3.4,$$

(adjusted *R*-squared 0.77). The KSS-model fit for days 9–11 of the sleep extension protocol is shown in Figure 5a. Model fits for the full 12 days of the protocol for both sleep extension and sleep restriction are given in Supplementary Figure S5a.

The modeled KSS consists of a linear combination of homeostatic and circadian components. These separate components are plotted in units of KSS in Figure 5a and indicate that daily circadian variation contributed ±1.5 KSS points and homeostatic sleep pressure contributed on average 0–2 KSS points, rising to three points after 40 h of wakefulness.

### Mathematical model simulation of the field protocol predicted KSS time course and sleep timing

Average simulated sleepiness increased across the evening but compared with field observations started from a lower value (3.2 KSS points (model); 3.9 KSS points (field)), had a smaller gradient (0.4 KSS points/h (model); 0.9 KKS points/h (field)) and had a smaller standard deviation at each point (average across the 9 time points: 0.11 KSS points (model); 1.06 KSS points (field)), see Figure 5b. The distribution of simulated evKSS8 measurements of all participants and all days also had a lower average and smaller range than field observations (Figure 5c).

In contrast, distributions of sleep durations and sleep onset times closely matched field observations and had a similar average, standard deviation and range (Figure 5c).

We did not measure circadian phase in the field. However, since circadian phase was an output of the model, for completeness the distribution of predicted circadian phases is shown in Supplementary Figure S6.

### Mathematical model predicted KSS correlated with bedtime, bedtime with subsequent sleep duration, sleep duration with next morning sleepiness, morning sleepiness with evening sleepiness

**Figure 6.**
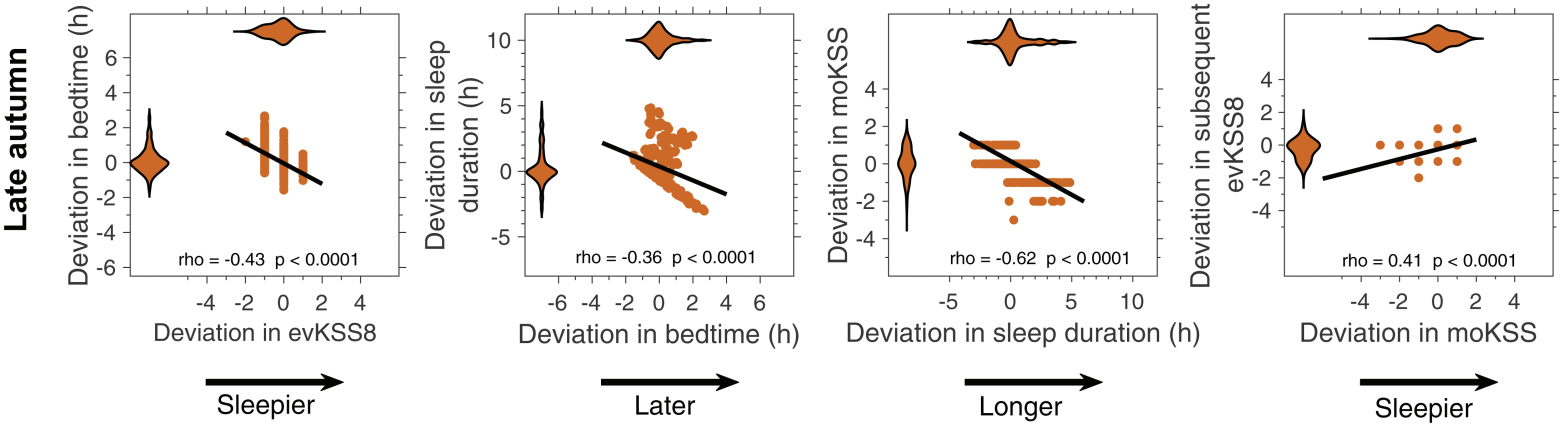
Mathematically modeled associations between evening sleepiness and subsequent bedtime, bedtime and subsequent sleep duration, sleep duration and the following morning KSS, morning KSS and the following evening KSS. For each panel the distributions of the correlated variables are shown using violin plots, where the violin plot for the variable plotted on the horizontal axis is at the top of the panel and the violin plot for the variable plotted on the vertical axis is to the left of the panel. Some of the violin plots suggest a multimodal distribution which relates to differences between weekdays and weekends. All variables are expressed as deviations from the participant median value. The Spearman rho correlation coefficients and associated p values are shown in each panel.

As observed in the field data, simulated deviations in evening sleepiness were significantly associated with simulated deviations in bedtime, simulated deviations in bedtime were significantly correlated with subsequent sleep duration, simulated deviations in sleep duration were significantly associated with next morning sleepiness and simulated next morning sleepiness was associated with subsequent evening sleepiness (Figure 6). Results are presented in the same way and on the same scales as the field observations shown in Figure 4 to facilitate comparison. Only the late autumn simulations are shown, the late spring simulations are similar. The model predicted correlations are in the same direction but tend to be higher than those observed in the field, see Supplementary Table S3.

## Discussion

Our study findings show that, regardless of individual differences in habitual sleep timing or sleepiness, higher than usual evening sleepiness associated with earlier than usual bedtime, longer than usual sleep duration, and lower than usual morning and following evening sleepiness. These associations were maintained over seasons and over bedtime categories. Associations between evening sleepiness and bedtime were more robust in early than in late sleepers.

### Sleepiness and the drive for sleep

The data are in accordance with the concept that sleep is a homeostatically-regulated motivated behavior as conceptualized in the 1960s and that self-reported sleepiness reflects the drive for that behavior. Subsequently this concept was complemented with a circadian view of sleep regulation [54] and ultimately these twin concepts were combined in the two-process model of sleep regulation [55].

A basic homeostatic principle is that deviations from equilibrium will be corrected. Thus higher than usual sleepiness should result in an increased drive for sleep, which should lead to preparatory behaviors followed by sleep initiation. Our results show that sleepiness assessed as long as 4 h before habitual bedtime is already predictive of sleep timing, with day-to-day variations in sleepiness predictive of corresponding day-to-day variations in bedtime. A later bedtime leads to shorter sleep duration because of the circadian regulation of sleep termination and social constraints, both of which determine wake time. Shorter sleep duration results in sleep debt which is reflected in increased subjective sleepiness the next day, which in turn will lead to earlier bedtime and ultimately the re-establishment of homeostatic equilibrium.

In this framework, reduced perceived sleepiness in the presence of physiological sleep need, is a cause of sleep deprivation.

### Time course of sleepiness

Under the natural conditions of this study, sleepiness built up gradually towards bedtime, as seen in Figures 3 and 5, and average values were consistent with the average of 4.1 (±1.3 *SD*) KSS points reported by others [13]. Furthermore, the average time course was consistent with other field studies in which sleepiness was plotted for week days and days off against clock time or relative to habitual bedtime [13]. This time course is, however, somewhat different from those observed in laboratory studies and in particular constant routine protocols in which sleepiness was plotted relative to habitual wake time, bedtime or circadian phase [7, 14]. In these studies, in which participants’ activity, workload, posture, food intake and light exposure was near constant throughout the wake period, sleepiness did not markedly increase in the period before habitual bedtime. Furthermore, sleep onset typically occurred at around five KSS points in laboratory studies and seven KSS points in the field.

These differences between field and laboratory then present a mathematical modeling challenge. KSS is not a direct output of the mathematical model. We used the model output “sleep drive” as a proxy for KSS. Since our aim was to see if current models could replicate findings in the field without fitting, we elected to scale sleep drive according to the laboratory protocol and accept that this would necessarily mean that modeled KSS values in our simulation of the field study would be lower than field observations. These differences in scaling can account for the smaller slope of KSS across the evening and the lower average value of evKSS8 found in the simulation as compared with the field observations.

Causes underlying these different time courses in the laboratory and field remain unclear [2]. One explanation is that participants in the field study are more sleep deprived than in the laboratory studies. Understanding the causes underlying these differences is important for the development of models for sleepiness in real world situations, such as shift work.

### Intraindividual variation in sleepiness

The most surprising aspect of the evening sleepiness data was the very large variation from day-to-day. At the same time point, 4 h before habitual bedtime, some individuals rated themselves very alert (KSS = 2) on some days and very sleepy, great effort to keep awake (KSS = 9) on others. Most individuals varied their KSS scores by four or five points, assessed at the same time point on different days.

These large evening-to-evening variations in KSS are unlikely to be related to large variations in circadian phase because circadian phase is remarkably stable from day-to-day even over the weekends. Thus, in an assessment of circadian melatonin phase before and after a weekend (two nights) during which sleep onset was on average delayed by 1.5 h and sleep offset delayed by 3 h, melatonin phase was delayed by only 45 min [56]. Consistent with these observations, our model simulations (see Figure 2, right hand panel) suggested that daily changes in light accounted for variations in phase of only 1 h. In fact, when one models the KSS as the sum of a circadian and homeostatic process, laboratory data from forced desynchrony protocols [3, 57] and our simulations (Figure 5) suggest that variation in circadian phase can alter KSS by at most ±1.5 points.

Similarly, day-to-day changes in sleep homeostasis does not appear to be a plausible explanation for the large deviations observed. Laboratory data suggest that time awake contributes on average 0–2 KSS points, only rising to three points after 40 h of wakefulness (Figure 5). Markers of the homeostatic process such as slow wave activity (SWA) [58] and slow wave sleep (SWS) [59] remained unchanged following variations in cognitive load; although subjective ratings of tiredness [58] and both sleep latency and wake time after sleep onset [59] increased under higher loads.

Thus, the evening-to-evening variation in KSS appears to be related in part to prior sleep duration and “random” variation or behavioral factors (e.g. caffeine/alcohol/medications intake, workload, etc.). In the model this random variation was represented by “noise” which was added to the threshold for switching from wake to sleep and physiologically models “excitement,” “arousal,” direct effects of light, etc. Even then the simulated within participant variation in KSS was smaller than the variation in the data, which could not be accounted for by differences in scaling.

### Intraindividual variation in sleep timing

The fact that the associations between sleepiness and sleep timing were stronger in early than late sleepers is consistent with the view that within a particular age group early sleepers are more strongly influenced by homeostatic effects [60] and with data showing that morning types have more SWS and SWA [61, 62].

**Figure 7.**
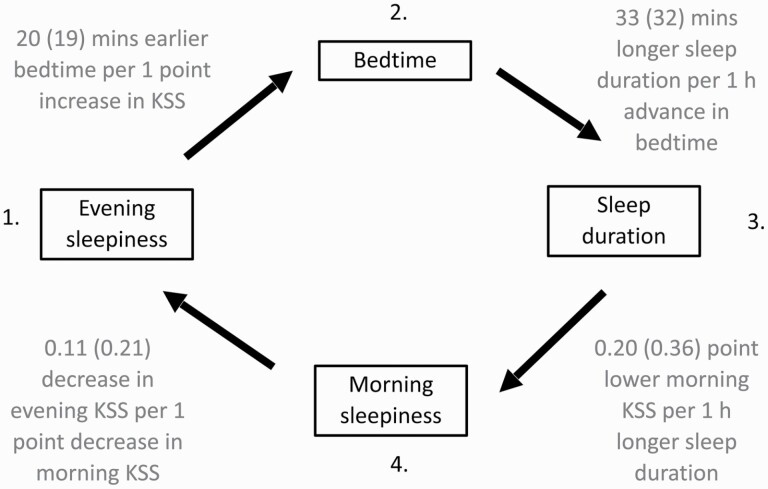
Summary of the homeostatically-regulated chain of events. Field data and simulations support a homeostatic chain of events where deviations from equilibrium are corrected. So higher evening sleepiness leads to earlier bedtime, earlier bedtime to longer sleep duration, longer sleep duration to lower morning sleepiness, lower morning sleepiness to lower evening sleepiness. Magnitudes of the associations are given for both the field data and the model, with those from the model given in brackets.

Our simulations quantitatively captured the average, variance and individual deviations in sleep duration and timing and predicted associations in the same direction as our field measurements (Figures 5 and 6, and summarized in Figure 7). The fact that the associations were typically stronger in the model than observed in the data is indicative that we have not sought to include further random effects associated with physiology, environmental and social factors. The simulations support the view that the associations seen in the data represent a causal homeostatic chain, with perturbations away from equilibrium subsequently being self-corrected.

We note though, that an interesting feature of the two-process model is that going to bed a few hours later is compensated by sleeping more deeply. Consequently, according to the model, shorter sleep duration only results in higher levels of next morning sleepiness if sleep is curtailed by social obligations and not if it is simply a result of going to bed at a later circadian phase.

### Role and effects of sleepiness

Our participants consistently reported sleepiness and tiredness as the leading determinant of their decision to go to bed. The results complement a recent laboratory study [63], that reported that following a single night of total sleep deprivation, increased sleepiness was associated with increased self-reported motivation for sleep preparatory behaviors such as rest and being alone and decreased motivation for physical and social activities. In a recent analysis of the association between sleepiness and social activity in the real world it was found that sleepiness was associated with reduced social activities in the evening in particular [64].

### Strengths and weaknesses of current study

The study strengths include repeated nightly measurements over two three-week periods and two seasons in the ecological environment. Although self-report measures may be considered a study limitation, the KSS is a widely used and well-validated instrument. KSS is well-established as the earliest and most sensitive responder to both partial, acute total sleep deprivation and selective SWS deprivation [7, 9–11]. The effect sizes that we observed were significant but small to medium, which leaves considerable unexplained variance. Our sample size was relatively small and we studied only one age group. Furthermore, the steep rise of sleepiness and its large variation during the evening remain unexplained both from a physiological/sleep regulatory and a modeling perspective. Further modeling refinements could have been made. For example, we modeled between individual differences by allocating different model participants different intrinsic circadian periods. This captured differences in sleep timing, but did not capture differences in sleep need or circadian amplitude. The strict constraint of insisting that the model participants woke at a fixed time during the week meant that going to bed later necessarily led to shorter sleep duration, independent of any circadian effect. The clear distinction between week days and weekends in the modeling accounts for the multimodality that is seen in the violin plots for sleep duration. In the data, the distinction between days of the week was more blurred.

### Implications

The associations we found between subjective sleepiness and bedtime, sleep duration and next day sleepiness imply that interventions that facilitate the awareness and perception of sleepiness may be beneficial. There are many environmental and behavioral factors which may impact sleepiness and thereby impact the decision to go to bed. Often cited factors are evening light exposure [65], caffeine [66], evening screen time [67] and social media use [68]. Some of these factors (e.g. light, caffeine) may bring about their effects on sleep timing through their effects on sleepiness [23, 24, 69]. The magnitude of the suppressive effects of light [24] and caffeine [70] on sleepiness are in the range of 1–1.5 KSS which, according to the current data, would lead to 20–30 min later bedtime.

## Conclusions

Our study findings showed that higher than usual evening sleepiness associated with earlier bedtime, which associated with longer sleep duration, and lower morning sleepiness. These associations were apparent in both seasons and in general were stronger in individuals with early sleep timing compared to those with late sleep timing. Data and model simulations showed that sleepiness was a significant determinant of the decision to go to bed, and suggest that sleepiness serves as an important signal for maintaining stable sleep patterns. However, the discrepancies between the data and model simulations and the discrepancies between the time course of KSS in laboratory and field studies indicate that our understanding of the determinants of KSS remains limited.

Further experiments in which high frequency and repeated assessments of KSS, as in this study, are combined with detailed descriptions of daily activities, light exposure, caffeine consumption and nocturnal sleep physiology are needed to provide new insights in the regulation of sleepiness and sleep.

## Supplementary Material

zsab123_suppl_Supplementary_Materials

## Data Availability

The data underlying this article will be shared on reasonable request to the corresponding author.
