# Supplementary material for: Sleepiness is a signal to go to bed: data and model simulations
Source: Sleep. 2021 May 15;44(10):zsab123. doi: 10.1093/sleep/zsab123 (PMC8503825; doi:10.1093/sleep/zsab123)
Supplement: zsab123_suppl_Supplementary_Materials [file zsab123_suppl_supplementary_materials.pdf]

Sleepiness is a signal to go to bed: data and model simulations

Supplementary Material

Tamar Shochat[1], Nayantara Santhi[2,3] Paula Herer[1],

Derk-Jan Dijk[2,4,†], Anne C Skeldon[5,4,†,*]

[1] Cheryl Spencer Department of Nursing,
Faculty of Social Welfare and Health Sciences,
University of Haifa, Haifa, Israel
[2] Surrey Sleep Research Centre, Faculty of Health and Medical Sciences,
University of Surrey, Guildford, GU2 7XP, UK
[3] Department of Psychology, Faculty of Health and Life Sciences,
Northumbria University, Newcastle Upon Tyne, UK
[4] UK Dementia Research Institute Care Research & Technology Centre,
at Imperial College London and the University of Surrey, Guildford, UK
[5] Department of Mathematics, Faculty of Engineering and Physical Sciences,
University of Surrey, Guildford, GU2 7XH, UK

[†] These authors contributed equally to the manuscript

[*] Coresponding author: a.skeldon@surrey.ac.uk

June 3, 2021

1 Protocol adhesion

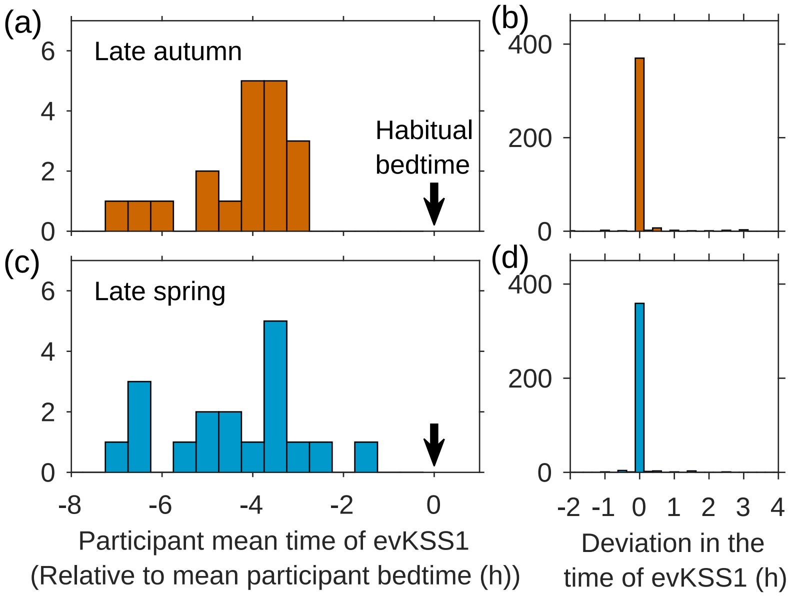

Figure S1: **KSS completion times** (a),(c) Mean time of the first evening KSS (evKSS1) relative to mean participant (autumn) bedtime; (b),(d) Histogram for late autumn and late spring showing of the times of the participants' individual nightly recordings for the first evening KSS, evKSS1, relative to the participant mean. This shows that participants reliably completed their KSS recordings at the same times each day.

2 Associations by day-of-the-week

Day	Association	n	rho	p	Fisher Z, p*
Sun	evKSS8 - Bedtime	99	-0.26	0.011	0.07, 0.47
Mon	evKSS8 - Bedtime	95	-0.27	0.007	-0.37, 0.36
Tue	evKSS8 - Bedtime	102	-0.22	0.027	1.15, 0.13
Wed	evKSS8 - Bedtime	98	-0.37	$<$0.001	2.05, 0.02
Thu	evKSS8 - Bedtime	97	-0.09	0.397	1.53, 0.06
Fri	evKSS8 - Bedtime	104	-0.30	0.002	0.23, 0.41
Sat	evKSS8 - Bedtime	100	-0.33	0.001	-0.53, 0.30
Sun	Bedtime - Sleep duration	104	-0.38	$<$0.0001	0.88, 0.19
Mon	Bedtime - Sleep duration	105	-0.48	$<$0.0001	0, 0.5
Tue	Bedtime - Sleep duration	106	-0.48	$<$0.0001	-1.54, 0.06
Wed	Bedtime - Sleep duration	108	-0.30	0.0015	0.74, 0.23
Thu	Bedtime - Sleep duration	106	-0.39	$<$0.0001	0.25, 0.40
Fri	Bedtime - Sleep duration	99	-0.42	$<$0.0001	1.29, 0.10
Sat	Bedtime - Sleep duration	101	-0.56	$<$0.0001	-1.64, 0.05
Sun	Sleep duration - moKSS	102	-0.19	0.056	0.22, 0.41
Mon	Sleep duration - moKSS	103	-0.22	0.023	0, 0.5
Tue	Sleep duration - moKSS	105	-0.22	0.022	-0.38, 0.35
Wed	Sleep duration - moKSS	104	-0.27	0.007	0.67, 0.25
Thu	Sleep duration - moKSS	104	-0.18	0.069	0.15, 0.44
Fri	Sleep duration - moKSS	97	-0.20	0.044	1.13, 0.13
Sat	Sleep duration - moKSS	101	-0.35	$<$ 0.001	-1.22, 0.11
Sun	moKSS - evKSS8	100	0.12	0.229	-
Mon	moKSS - evKSS8	79	-0.06	0.602	-
Tue	moKSS - evKSS8	97	0.08	0.448	-
Wed	moKSS - evKSS8	95	0.13	0.197	-
Thu	moKSS - evKSS8	91	0.06	0.603	-
Fri	moKSS - evKSS8	99	0.12	0.236	-
Sat	moKSS - evKSS8	91	0.17	0.111	-

Table S1: **Spearman rho correlations between intra-individual variation in evening sleepiness and subsequent bedtime, bedtime and subsequent sleep duration, sleep duration and subsequent morning sleepiness, and morning sleepiness and subsequent evening sleepiness, by day-of-the-week.** All variables were expressed as deviations from the median per participant per season and then entered into the correlation analysis. evKSS8: eighth evening KSS; moKSS: next morning KSS. Fisher Z tests report comparisons between successive days such that the first entry is the comparison between Sunday and Monday etc.

.

3 Mathematical modelling: Equations and modelling assumptions

The results shown in Fig. 4 come from numerically integrating a mathematical model for sleep wake regulation using the stiff solver ODE15s in MATLAB [1]. The model is a neuronal model that includes sleep homeostasis, circadian rhythmicity, light [2] and social constraints [3]. Here, we have in addition added some randomisation to capture within and between subject variations.

Full equations, parameter choices and details of the randomisation are given below.

3.1 Equations

As in [2, 3], sleep/wake regulation was modelled as the interaction between sleep promoting and wake promoting neurons as described by equations for their mean electric potential, V_v and V_m, respectively,

$$\tau \frac{dV_v}{dt} = -V_v - \nu_{vm} Q_m + D_v, \quad (1)$$
$$\tau \frac{dV_m}{dt} = -V_m - \nu_{mv} Q_v + D_m. \quad (2)$$

Here,

$$Q_{v,m} = \frac{Q_{\max}}{1 + \exp[-(V_{v,m} - \theta)/\sigma]}, \quad (3)$$

describes the sigmoidal relationship between the potential and the firing rate of the neurons, $Q_{v,m}$. The parameter $Q_{\max}$ is the maximum possible firing rate; θ is the value of the potential $V_{v,m}$ at $Q_{v,m} = Q_{\max}/2$ and σ determines the width of the sigmoid.

The parameter τ gives the typical timescale of the neuronal process and the parameters $\nu_{vm,mv}$ weight the input from population m to v and v to m respectively.

The $D_{v,m}$ are 'drives'. The drive to the wake promoting neurons, D_m was fixed. The drive to the sleep promoting neurons, D_v, consisted of homeostatic and circadian components,

$$D_v = A_v - \nu_{vc} C(t) + \nu_{vh} H(t), \quad (4)$$

where $H(t)$ describes the homeostatic sleep pressure and $C(t)$ represents the circadian wake promoting rhythm. As discussed further below, $C(t)$ is approximately sinusoidal with amplitude close to one, so ν_{vc} gives the amplitude of the circadian wake propensity rhythm.

The term A_v is a background excitatory input to the sleep promoting neurons. In previous work, A_v has been kept fixed. Here, A_v has a time course which fluctuates around a mean to capture minute-by-minute changes in stimulation. Further details are given below.

The homeostatic process is given by

$$\chi \frac{dH}{dt} = -H + \mu Q_m. \tag{5}$$

Here, H/χ is the rate of removal of somnogenic chemical and $\mu Q_m/\chi$ is the rate of production.

The circadian rhythm $C(t)$ was modelled by a forced van der Pol oscillator [4], where the forcing represents the light intensity dependent signal to the suprachiasmatic nucleus from photoreceptors in the eye. The model was originally constructed to accurately replicate human phase response data [4]. Specifically,

$$\kappa \frac{dx}{dt} = \gamma \left(x - \frac{4x^3}{3} \right) - y \left(\left(\frac{24}{f\tau_c} \right)^2 + kB \right), \tag{6}$$

$$\kappa \frac{dy}{dt} = x + B, \tag{7}$$

$$\frac{dn}{dt} = \lambda \left(\alpha_0 \left(\frac{\tilde{I}}{I_0} \right)^p (1-n) - \beta n \right), \tag{8}$$

where

$$B = \alpha_0 G(1-n)(1-bx)(1-by) \left(\frac{\tilde{I}}{I_0} \right)^p, \tag{9}$$

$$\tilde{I} = \mathcal{H}(Q_m - Q_{th}) I(t). \tag{10}$$

Here, x and y are the variables for the van der Pol oscillator and n is the fraction of activated photoreceptors. The term B describes the forcing that occurs as a result of the action of light of intensity I on photoreceptors. The magnitude of B is dependent on phase, as modelled by the term $(1-bx)(1-by)$ in equation (9). The rate of saturation of photoreceptors is given by $\lambda\alpha_0 \left(\frac{\tilde{I}}{I_0}\right)^p$ and the rate for decay is given by $\lambda\beta$, where the values of $\lambda, \alpha_0, I_0, p$ and β have been determined from experimental data [5]. The parameter τ_c is the intrinsic period, γ is the stiffness of the oscillator.

Note that the intrinsic period of the van der Pol oscillator was dependent on light intensity since for diurnal species intrinsic period is shorter for higher light intensities (Aschoff's rule). This dependence of the intrinsic period on light intensity is controlled by the parameter k in equation (6).

As in [3], the circadian wake propensity rhythm $C(t)$, was modelled as

$$C(t) = \frac{1}{2}\left(1 + 0.80y - 0.47x\right). \tag{11}$$

The Heaviside function in equation (10), means that light impinging on photoreceptors was zero if the firing rate of wake promoting neurons, Q_m, is greater than a threshold value Q_{th}. This models gating of light by sleep.

**Sleep/wake regulation parameters**					
$Q_{\max}$	100 s^{-1}	θ	10 mV	σ	3 mV
ν_{vm}	2.1 mVs	ν_{mv}	1.8 mVs	ν_{vc}	3.84 mV
ν_{vh}	1 mVnM^{-1}	A_m	1.3 mV	A_v	-10.2 mV
τ	10 s			χ	$45 \times 60 \times 60$ s
μ	4.41 nMs	Q_{th}	1 s^{-1}		
**Circadian parameters**					
κ	$\frac{12}{\pi} \times 60 \times 60$ s	γ	0.23	f	0.99669
τ_c	$N(\bar{\mu}, \sigma^2), \bar{\mu} = 24.2$ h, $\sigma = 0.15$ h	k	0.55	α_0	0.16
G	19.9				
I_0	9500 lux	β	0.013	b	0.4
p	0.6				
**Light parameters**					
c	$1/6000$	l_1^i	log normal distribution	l_2	35 lux
s_1	$10.5 \times 60 \times 60$ s	s_2	$15.5 \times 60 \times 60$ s (late autumn)		
		s_2	$17.5 \times 60 \times 60$ s (late spring)		

Table S2: **Parameter values**

All the parameters values used are listed in Table **S2**. The sleep/wake regulation and circadian parameters are the same as those used in the original version of the model [2]. A typical solution for equations (1)-(11) is given in Fig. **S2**.

3.2 Social constraints

In the late autumn sleep diary, the alarm clock question was open-ended: Did your alarm clock wake you up?

Whenever the participant answered that they did, it was obvious that the alarm clock was set. In instances where they answered in the negative and further gave a specific reason such as ‘I did not set the alarm’ or ‘no I woke up xx minutes before the alarm’, it was also clear whether the alarm was set or not. In instances where this was not the case it was not possible to infer whether or not the alarm was set.

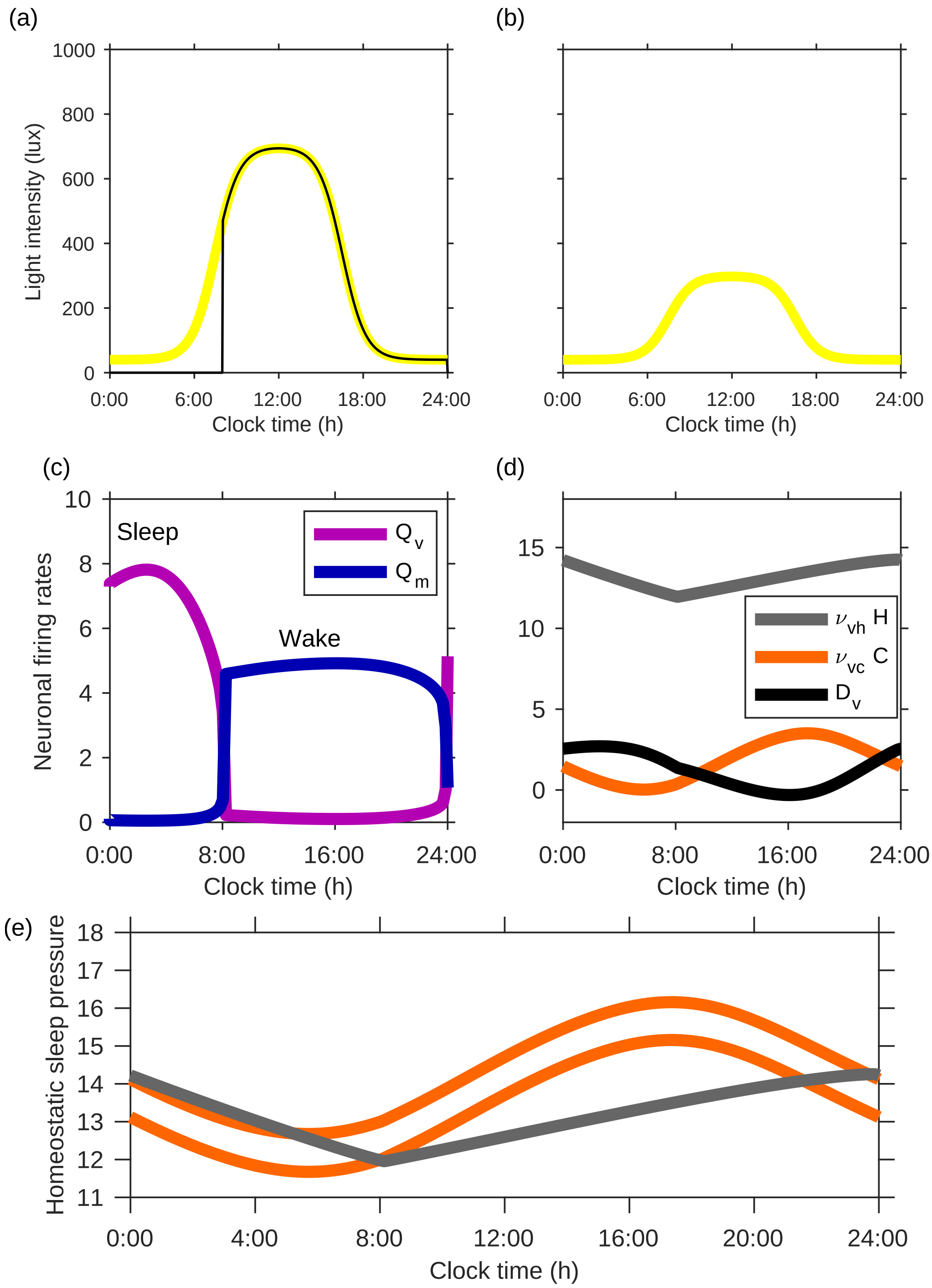

Figure S2: **Typical model inputs and outputs (without noise)** (a) and (b) typical light profiles (yellow) given by equation (15) for $l_1 = 700, 300$ respectively; $l_2 = 40$ in both cases. All other parameters are given in Table **S2**. In (a), the thin black line shows the light profile gated by the sleep-wake cycle for the particular case shown in (c)-(e). (c) The firing rates for the wake promoting and sleep promoting neurons, Q_m and Q_v respectively. (d) The homeostatic sleep drive, $\nu_{vh}H$ (dark grey) and the circadian wake propensity $\nu_{vc}C$ (orange). The difference between these two components gives the sleep drive D_v (black). (e) The data re-plotted in the form of the two process model, showing the homeostatic sleep presssure decreasing during sleep until it reaches the lower threshold, then increasing during wake until spontaneous sleep occurs when the homeostatic sleep pressure reaches the upper threshold. The upper and lower thresholds are modulated by the circadian wake propensity rhythm. For (c)-(e), the light profile illustrated in (a) was used; $\mu = 4.20, \nu_{vc} = 3.37, \tau_c = 24.2$. All other parameter values are given in Table **S2**. This figure is reproduced from the Supplementary Material for [3].

In the Spring sleep diary, the alarm clock question was changed from an open-ended to forced choice: Did your alarm clock wake you up? YES/NO. An additional question was added at the beginning of the sleep diary: Did you set an alarm? YES/NO.

Together, this indicated that on 88% of weekdays and 48% of weekend days students set an alarm to wake up, see Fig. **S3**.

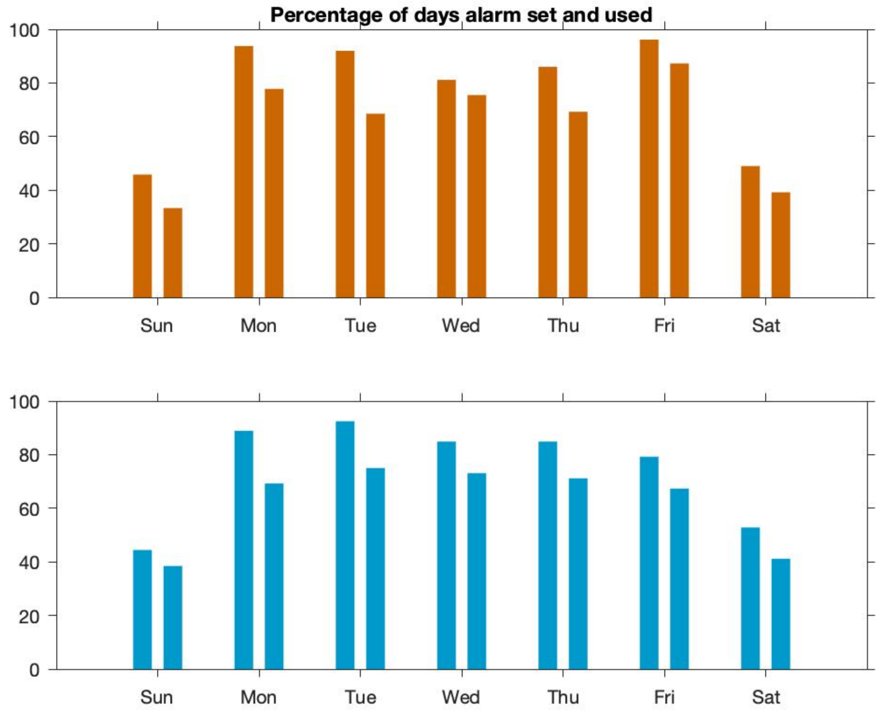

Figure S3: **Percentage of days the alarm was set and used**. The top panel shows the autum and the bottom panel the spring. For each day, the left hand column indicates the percentage of the time the alarm was set and the right hand column the percentage of days the alarm was used.

Motivated by the fact that participants mostly set an alarm during the week, and do not set an alarm at the weekend for each 'participant' we construct a repeating seven day schedule. On the five week days the model was required to be awake at or before a fixed alarm time and to stay awake until at least 19:00 h. On weekend days there were no constraints.

Full details of how we model social constraints were previously given in the Supplementary Material to [3], where we also explained the underlying dynamics. Below we reproduce the key elements of the resulting algorithm.

On days when there were social constraints, the following procedure was followed. The

model was integrated until the alarm time. There are then three scenarios

(i) $D_v < D_V^-$. The model is already in the wake state, so integration is continued as normal.

(ii) $D_v^- < D_v < D_v^+$, then D_v is in the hysteretic region where both sleep and wake are possible states. A switch from sleep to wake is therefore made by instantaneously increasing V_m and decreasing V_v. The equations are then integrated from the new initial conditions.

(iii) $D_v > D_v^+$, then no wake state exists. However, a 'forced' wake state can be reached by increasing the drive to the wake promoting neurons. Specifically, for values of $D_v > D_v^+$ we solve the circadian oscillator equations (6)-(9) along with

$$\chi \frac{dH}{dt} = -H + \mu Q_m(D_m^+, D_v), \tag{12}$$

where $Q_m(D_m^+, D_v)$ is the firing rate of the wake promoting neurons at the saddle-node bifurcation denoting the change from wake to sleep. The value of $Q_m(D_m^+, D_v)$ is given by the solution of

$$\begin{aligned} D_v &= V_v - \nu_{vm} Q_m \\ D_m^+ &= V_m - \nu_{mv} Q_v \\ \frac{\nu_{vm}\nu_{mv}}{\sigma^2} &= (Q_v - Q_{\max})(Q_m - Q_{\max}), \end{aligned} \tag{13}$$

where

$$Q_{v,m} = \frac{Q_{\max}}{1 + \exp[-(V_{v,m} - \theta)/\sigma]}. \tag{14}$$

The forced wake state is continued until $D_v = D_v^+$, and the procedure outlined under (ii) is followed. This is equivalent to the method described in [6] for following the so-called wake 'ghost' during sleep deprivation.

If the model switches to sleep during the period after the alarm but before 19:00 h, the forced wake regime is again implemented.

3.3 Randomisation

3.3.1 Physiological: Intrinsic circadian period

We assigned each of our model population of 18 'participants' a different intrinsic circadian period. Specifically, intrinsic circadian period, τ_c was assumed to be normally distibuted with a mean of 24 h and a standard deviation of 0.15 h, consistent with [7].

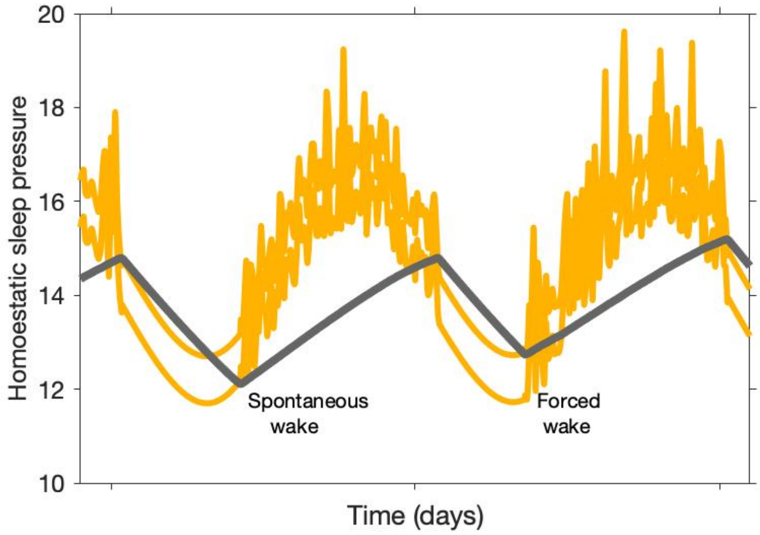

Figure S4: **Example of noisy upper thresholds**. Two-process representation of the model, showing how noicse noise added to the sleep drive, D_v results in varying in the upper threshold position during wake. Two days are shown. On the first day, spontaneous wake occurs when the homeostatic sleep pressure (grey line) hits the lower threshold (lower orange line). On the second day, wake is 'forced' by the social constraint, so wake occurs before sleep homeostatic sleep pressure has dissipated.

3.3.2 Physiological: Momentary wake propensity

Minute-by-minute changes in wake propensity due to stimulation such as caffeine, excitement or relaxation were modelled as changes to the sleep drive. Specifically, we changed the drive to sleep promoting neurons A_v by adding a random number A_v^{noise} that is drawn from a log normal distribution.

$$A_v^{\text{noise}} = -\text{lognrnd}(0.1, 0.5) + 0.5.$$

With the rationale that this is a process on the scale of a few minutes, we have changed A_v every 5 minutes, linearly interpolating between neighbouring values.

We have made the modelling assumption that A_v returns to baseline during sleep.

Adjusting A_v in this way is equivalent to simultaneously shifting both the upper threshold and lower threshold. An example illustrating the resulting 'two process' dynamics is shown in Fig. **S4**.

3.3.3 Environmental: Light signal

Motivated by the shape of real world light profiles [8, 9], we have used the function

$$I(t) = l_2 + \frac{(l_1^i - l_2)}{2} \left(\tanh\left(c(\text{mod}(t, 24 \times 60 \times 60) - s_1)\right) - \tanh\left(c(\text{mod}(t, 24 \times 60 \times 60) - s_2)\right)\right). \tag{15}$$

This switches between a level l_1^i during core day light hours on day i and l_2 at other times, where the switch from l_2 to l_1 occurs around $t = s_1$ and from l_1 to l_2 around $t = s_2$. The speed of the switch is determined by the parameter c.

In order to capture day-to-day variability in the amount of light received, the value of l_1^i was drawn from a log normal distribution. Seasonal differences were modelling by setting a lower mean value for the late autumn than the late spring and by changing the value of s_2.

4 Modelling sleepiness

The sleep drive D_v is a measure of the sleep propensity rhythm. By fitting to data from a laboratory sleep extension/restriction with subsequent sleep deprivation protocol [10] a scaling between D_v and sleepiness as measured on the Karolinska Sleepiness Scale (KSS) was constructed.

The protocol consisted of a habituation night, a baseline night, then 7 further nights on which sleep opportunity was either restricted to 6 h time in bed or extended to 10 h time in bed. Following the last night of sleep participants underwent a constant routine of 39 h (sleep extension condition) or 41 h (sleep restriction condition). KSS was evaluated at regular intervals during scheduled wake times. The study had a balanced cross-over design and 36 young adults took part. Further details on the protocol can be found in [10].

Here, we averaged KSS values across all participants at each of the measurement times, resulting in 123 data points spread at regular intervals across the 12 days of the prototcol (61 during SE, 62 during SR). We then simulated the protocol and found the value of D_v for each of the times that laboratory assessments were made. The linear best fit line between D_v and KSS gave

$$\text{KSS} = 0.79 D_v + 3.4. \tag{16}$$

The averaged data and the fitted time course are shown in Fig. **S5**(a). KSS versus D_v and the linear regression line are shown in Fig. **S5**(b).

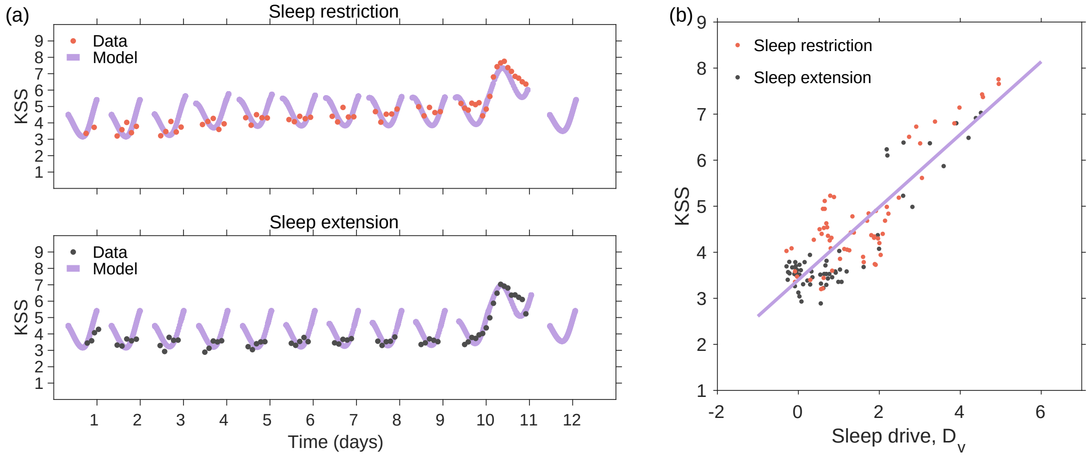

Figure S5: **Model fits to the sleep extention / sleep restriction with subsequent sleep deprivation protocol**. (a) Time course of average KSS observations and fitted sleep drive for the two conditions. (b) Average KSS observations versus sleep drive D_v.

5 Further model outputs from simulation of the field protocol

5.1 Circadian phase distribution in simulations of the field protocol

Circadian phase is an output of the simulations of the field protocol. The distributions of circadian phase are shown in Fig. S6.

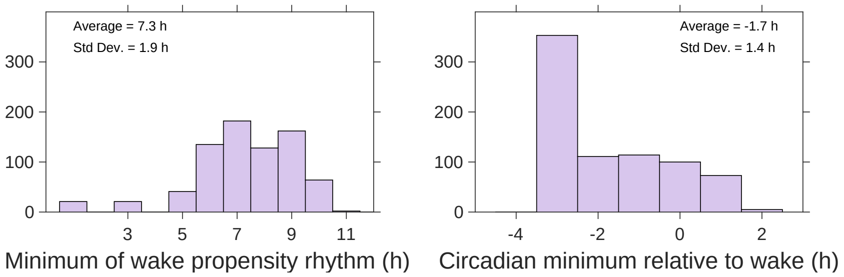

Figure S6: **Circadian phase from simuation of the field protocol**. Left hand panel: Predicted distribution of the circadian wake propensity minimum (clock time). Right hand panel: Predicted distribution of the circadian wake propensity minimum (relative to wake).

5.2 Comparison between associations from field observations with those deduced from simulations of the protocol

	Field data		Model	
	Late autumn	Late spring	Late autumn	Late spring
BT- evKSS8 (min / KSS point)	-22.80	-14.40	-35.40	-10.20
BT - SD (min / h)	-32.40	-33.00	-31.80	-48.00
SD - moKSS (KSS point / h)	-0.26	-0.18	-0.36	-0.48
moKSS - evKSS8 (KSS point/ KSS point)	0.06	0.13	0.30	0.08

Table S3: **Linear regression slopes for the associations between deviations from individual participant median.**

References

[1] The Mathworks, Inc., Natick, Massachussets. *MATLAB version (2019a)*, 2019.

[2] A.J.K. Phillips, P.Y. Chen, and P.A. Robinson. Probing the mechanisms of chronotype using quantitative modeling. *J. Biol. Rhythms*, 25:217–227, 2010.

[3] A.C. Skeldon, A.J.K. Phillips, and D.-J. Dijk. The effects of self-selected light-dark cycles and social constraints on human sleep and circadian timing: a modeling approach. *Sci. Rep.*, 7:45158, 2017.

[4] D.B. Forger, M.E. Jewett, and R.E. Kronauer. A simpler model of the human circadian pacemaker. *J. Biol. Rhythms*, 14:533–537, 1999.

[5] R.E. Kronauer, D.B. Forger, and M.E. Jewett. Quantifying human circadian pacemaker response to brief, extended and repeated light stimuli over the phototopic range. *J. Biol. Rhythms*, 14:501–515, 1999.

[6] B.D. Fulcher, A.J.K. Phillips, and P.A. Robinson. Modeling the impact of impulsive stimuli on sleep-wake dynamics. *Phys. Rev. E*, 78:051920, 2008.

[7] J.F. Duffy, S.W. Cain, A.M. Chang, A.J.K. Phillips, M.Y. Münch, C. Gronfier, J.K. Wyatt, D.-J. Dijk, K.P. Wright, and C.A. Cziesler. Sex difference in the near-24-hour intrinsic period of the human circadian timing system. *Proc. Natl. Acad. Sci. U. S. A*, 108:15602–15608, 2011.

[8] H.C. Thorne, K.H. Jones, S.P. Peters, S.N. Archer, and D.-J. Dijk. Daily and seasonal variation in the spectral composition of light exposure in humans. *Chronobiol. Int.*, 26:854–866, 2009.

[9] K.P. Wright, A.W. McHill, B.R. Birks, B.R. Griffin, T. Rusterholz, and E.D. Chinoy. Entrainment of the human circadian clock to the natural light-dark cycle. *Curr. Biol.*, 23:1554–1558, 2013.

[10] J.C. Lo, J.A. Groeger, N. Santhi, E.L. Arbon, A.S. Lazar, S. Hasan, S.M. von Schantz, S.N. Archer, and D.-J. Dijk. Effects of partial and acute total sleep deprivation on performance across cognitive domains, individuals and circadian phase. *PLoS ONE*, 7:e45987, 2012.
